# Computer security for data collection technologies^☆^

**DOI:** 10.1016/j.deveng.2017.12.002

**Published:** 2018

**Authors:** Camille Cobb, Samuel Sudar, Nicholas Reiter, Richard Anderson, Franziska Roesner, Tadayoshi Kohno

**Affiliations:** ICTD Lab and Computer Security & Privacy Research Lab Computer Science & Engineering, University of Washington, USA

**Keywords:** ICTD, Data collection, Security

## Abstract

Many organizations in the developing world (e.g., NGOs), include digital data collection in their workflow. Data collected can include information that may be considered sensitive, such as medical or socioeconomic data, and which could be affected by computer security attacks or unintentional mishandling. The attitudes and practices of organizations collecting data have implications for *confidentiality*, *availability*, and *integrity* of data. This work, a collaboration between computer security and ICTD researchers, explores security and privacy attitudes, practices, and needs within organizations that use Open Data Kit (ODK), a prominent digital data collection platform. We conduct a detailed *threat modeling* exercise to inform our view on potential security threats, and then conduct and analyze a survey and interviews with technology experts in these organizations to ground this analysis in real deployment experiences. We then reflect upon our results, drawing lessons for both organizations collecting data and for tool developers.

## 1 Introduction

Technology has become an important tool for many non-governmental organizations (NGOs) and groups collecting data in the developing world. For example, technology can provide people in remote regions with access to financial services and allow organizations to collect vital information within communities they serve. Information and Communication Technology for Development (ICTD) is the study of what technology can accomplish and how technology is used in such low-resource settings around the world. ICTD takes a broad definition of “low-resource”. Areas affected by poverty are frequently the focus of ICTD, but any setting where things like limited connectivity, unreliable power, or lightly skilled personnel conspire to create a unique technological landscape might be relevant to ICTD. Although there have been some efforts to study and address computer security and privacy risks with technologies in an ICTD environment, both on a case-by-case basis for specific technologies and from an academic perspective, e.g., (Ben-David et al., 2011, Corrigan-Gibbs and Chen, 2014, Reaves et al., 2015), the space of “computer security meets ICTD” is still in its infancy. We contribute to this space through insights into how to evaluate and address computer security risks in ICTD environments.

To provide a foundation for our insights, we choose to focus on a particular class of technologies—data collection toolkits—and, in particular, a specific, widely-used instance of such a technology: Open Data Kit (ODK). Data is crucial for many NGOs and researchers to monitor and evaluate deployments or interventions and report to donors on activities. For example, organizations might collect patient information during clinic visits, assess the prevalence of pests in rural farmland, or document infrastructure in need of repair. ODK allows digital forms to be created without deep technical expertise, and has been used as a platform by numerous organizations. By studying computer security risks with ODK, we are able to extract lessons for both ODK and other data collection deployments, as well as infer lessons for other new ICTD technologies.

This work is a collaboration between an ICTD research group and a computer security and privacy research group and leverages methodologies from both communities. For example, our threat model for data collection technologies (Section 4) is the result of a large *threat modeling* process (used in computer security) that involved many members from both groups. We augment that with surveys and semi-structured interviews, and leverage our past experiences (within our ICTD research group) in conducting deep investigations with key stakeholders (Sections 6, 7). Our threat model provides an analytic overview of the potential issues for data collection technologies, and the surveys and interviews provide a context within which to appropriately interpret and evaluate the risks of threats that we identified.

**Contributions.** Our contributions are three-fold. First:

• *Threat Model.* We develop a threat model for ODK and other data collection systems.

Our threat model provides a broad, encompassing view of the *possible* threats to an ODK-like system, including possible adversaries and adversarial methods. However, computer security is not a binary property and just because a computer security attack *might* be possible does *not* mean that it is likely to happen in practice. Hence, a more nuanced approach to computer security is to not only identify the possible threats, but to understand the broader context for a deployed system. Providing an informed, broader contextual analysis is our second key contribution:

• *Survey and Semi-structured Interviews.* We report on a survey and semi-structured interviews with ODK deployment architects.

We use the results of the survey and interviews to extract insights into how ODK deployment architects think about security. “Think about security” is intentionally broad; we consider, for example, not only how deployment architects perceive threats, but what defensive mechanisms they have deployed and why, what incidents they have encountered and how they responded, and so on. Finally:

• *Broader Synthesis and Recommendations.* We consider overarching implications and recommendations.

Among the key takeaways: as in the developed world, computer security of data collection platforms in the developing world is about risk management. Though our survey and interviews surfaced real threats and security concerns—particularly about data loss and erroneous or fake data—many of the threats we consider abstractly seem to have not yet manifested in practice for many of the deployments we studied. Hence, the current level of security seems arguably appropriate in today's environment, particularly given the practical tradeoffs faced in balancing security with other deployment goals. However, ICTD systems (and their data) may persist for many years, and the risks may change over time, making it important for organizations to proactively consider and revise their threat models.

## 2 Background and related work

**Terminology.** For this paper, when we say “security” we refer to computer security (i.e., cyber security) rather than to the global development sense of the word (e.g., “food security”). We were cautious with terminology both during our interviews and in our later evaluation of the interview results. Second, we note that the computer security community often use the terms “security and privacy” in unison, because security vulnerabilities can lead to privacy compromises; other communities have more nuanced usages of the term privacy. In this paper, we use the computer security community's interpretation of the term privacy.

**Security concerns for data collection.** In several studies, researchers have identified security concerns and drawn attention to the need to be more thoughtful about securing data collected in developing world contexts. Hussain (2013) gives a broad overview of the potential risks of mobile phone use in global development projects. She describes the sensitive types of data that organizations collect and references the legal code of several countries to argue that it is worth securing. These recommendations are good starting points for organizations employing digital data collection. Our work investigates how organizations already engaged in data collection approach and understand security, as well as the threats they have encountered and considered.

Other projects have identified and studied specific security concerns in digital data collection. Mobile devices in digital data collection projects are frequently not owned by the people entering the data. Instead projects will typically provision and distribute phones as part of a deployment. Schwartz et al. (2013) explore how this leads to non-prescribed, personal usage of the devices and how it can impact deployments. Our work reiterates that such usage is a real concern for many deployments. Data collectors can also be a source of inaccurate data. Birnbaum et al. explore methods to detect fabricated survey data using passive analysis (Birnbaum et al., 2013) as well as through measuring surveyor behavior (Birnbaum et al., 2012). Respondents in our study also consider data fabrication a threat, and many collect GPS coordinates or photographs in an effort to manually detect fabricated data.

Providing important insight to the context that informs how some data collectors think about data security, many groups performing digital data collection have experience with paper data collection. Parikh (2005) notes that paper can provide a sense of familiarity and security beyond that provided by a borrowed mobile phone. Ghosh et al. (2015) echo these findings, noting that paper passbooks can be kept in a specific location and thus secured. Our work adds depth to comparisons of paper and digital data collection by discussing how intuitions around paper affect digital security practices, as well as by showing that not all respondents consider paper more secure than digital collection methods.

The security concerns identified in these works demonstrate the need for a broad examination of existing and potential security issues in deployments conducting digital data collection.

**Advances in security protocols for data collection technologies.** Mancini et al. (Mancini and Mughal, 2011) proposed a new system of APIs for mobile data collection designed to prioritize security. This protocol is promising and considers many of the factors which make data collection in this context difficult (e.g., low budgets and use of feature phones, remote locations, and phone sharing among data collectors); however, the authors point out that the protocol has yet to be implemented and tested to determine whether it is computationally feasible, and it must also be tested in real deployments. Additionally, ODK's design already makes some assumptions that diverge from the premise of Mancini et al. In particular, ODK's design requires the use of (low-budget) smart phones for data collection. Gejibo et al. (2012) describe how low budget phones could securely store data during mobile data collection. In later work, Gejibo et al. (2013) describe how a cloud-based server could store data securely. Both these works focus on how encryption should be implemented and integrated into mobile data workflows. Our work aims a step before these projects. We try to understand how users of a modern digital data collection system think about and implement security, potentially guiding workflow and tool decisions. For the threat models our respondents are currently considering, we find that the usability of secure protocols is a larger barrier to security than is a need for new, more secure protocols.

**Security in other developing world contexts.** Several previous projects have considered how security threats manifest in developing world contexts beyond digital data collection. Ben-David et al. (2011) describe how end-users of technology (ranging from USB storage devices to mobile banking) in the developing world face security threats that differ from those in the developed world. They describe five factors which contribute to these differences, including the prevalence of text message banking and piracy in the developing world. More recently, security researchers have studied apps used for mobile banking in the developing world and found them rife with technical security vulnerabilities (Reaves et al., 2015). While similar problems are present in apps created for and used by people in the developed world, this work echoes the findings of (Ben-David et al., 2011) that the prevalence of these factors in the developing world increases the potential reach of their effects. Corrigan-Gibbs and Chen (2014), as well as Bhattacharya and Thies (2011), provide deeper analysis of security threats that can result from the prevalence of sharing content by USB; Corrigan-Gibbs and Chen (2014) propose a means of leveraging the prevalence of this type of USB use to propagate software updates rather than viruses. Our work focuses on a specific type of technology usage—digital data collection. In this context, we conclude that although the security issues do present somewhat differently, they do not represent a fundamentally novel class of problems to those in the developed world.

**Security for specific user groups.** Security and privacy researchers have studied specific user groups (e.g., Journalists (McGregor et al., 2015), parents and teens (Czeskis et al., 2010), older adults (Caine et al., 2012), and victims of partner violence (Matthews et al., 2017, Freed et al., 2017)) to understand their unique security practices and needs. Methods and lessons from these studies have informed our work. In particular, Elliott and Brody conducted a study of low-income New Yorkers (Elliott and Brody, 2015) and Elliott also reported on the sensitivities associated with conducting a user study focused on security with participants whose information may be especially sensitive (Elliott, 2016). Le Blond et al. (Le Blonde et al., 2014) report on the characteristics of targeted cyber attacks on an NGO. Although their aim was to better understand targeted attacks in general, this study provides relevant context for our work since many ODK deployments are conducted by or affiliated with NGOs.

## 3 Open Data Kit: background

We focus on Open Data Kit (ODK) as a prototypical data collection application used in developing regions. ODK was initially created in 2008 by researchers with the goal of providing a general purpose tool to facilitate data collection in ICTD contexts (Hartung et al., 2010). It has been widely adopted, used in at least 125 countries and installed on hundreds of thousands of devices (ODK Team, 2015). ODK allows digital forms to be created without deep technical expertise. It supports traditional text and multiple choice questions and leverages sensors on the devices to capture rich data types, including GPS locations and photographs. Factors contributing to its success include the fact that it is adaptable to a variety of settings and data domains and is free and open source (Gupta et al., 2013, Hartung et al., 2010, Brunette et al., 2013). There are two versions of ODK. The initial version, which we designate as ODK 1.x, is the most widely used. A new version, ODK 2.x, with extended functionality is under development and has been used in a small number of deployments. The two versions are sufficiently similar in both workflow and design that the distinction between them is not significant to our discussion.

Basic ODK setup involves: (1) creating an XML form for each questionnaire using a graphic user interface, (2) downloading the ODK mobile application to an Android device, (3) setting up an ODK (local or cloud) server using a simple installer, and (4) downloading the XML forms to the mobile devices. Then, the deployment involves: (1) filling out these forms on mobile devices, from which data being is to the SD card, (2) syncing to the server when there is a data connection, and (3) accessing/analyzing data on the server.

**Stakeholders.** Typical roles, or stakeholders, in an ODK deployment include: (1) donors and policy makers that fund or oversee the work but do not make specific technical decisions; (2) deployment architects that create the forms, administer deployments, and make technical decisions, possibly based on input from an ethics board or organizational policy; (3) enumerators that complete surveys on mobile devices; and (4) beneficiaries that provide data to enumerators. These are representative of the roles someone might have in a deployment, though not all roles are necessarily represented in every deployment, one person might take on more than one role, and each role may be filled by more than one person. Within a deployment, individuals in each of these roles may or may not be trusted or trustworthy and will likely have a wide range of technical skills.

## 4 ODK and computer security

Having summarized key properties of ODK's design, we now proceed with a deeper analysis of its computer security properties. We consider this *security analysis*, with roots in the *threat modeling* process common in the field of computer security, as a contribution to the ICTD community for two reasons. First, it surfaces key computer security threats and opportunities that we believe ODK deployment architects should consider. Second, it expands on other threat modeling work in ICTD (e.g., (Ben-David et al., 2011, Corrigan-Gibbs and Chen, 2014, Reaves et al., 2015)) and provides a data point for threats that might arise for ICTD applications.

Our security analysis in this section is done in a theoretical, abstract sense: we consider potential computer security threats that *may* arise in an ODK deployment. In practice, no system is completely secure, and computer security consists of a series of tradeoffs and risk management. It may be the case that some threats are worth defending against (because the cost or risk of compromise is high) whereas others are not. However, it is important to identify a wide array of possible threats in order to enable informed decisions about these tradeoffs. We consider a number of such threats in this section; our surveys and interviews in Sections 6, 7 then provide insights into the relative importance and likelihood of these risks and threats, as perceived by current ODK users.

### 4.1 Potential threats to an ODK deployment

Threat modeling is a process commonly used in the computer security community by which one identifies potential adversaries and their motivations, as well as potential threats and vulnerabilities. Our approach involved systematic brainstorming discussions and an empirical analysis of possible vulnerabilities in an archetypal ODK application (written specifically for this purpose). We performed brainstorming using Security Cards^2^ representing different assets, stakeholders, and adversaries, which we have found effective in the past at facilitating threat modeling. We present the results of our threat modeling exercise, stressing again that we made an effort to be thorough in identifying *potential* threats, and the issues we raise may or may not be faced by ODK deployment architects in practice.

**Security and privacy goals.** We begin by identifying possible security and privacy goals that stakeholders in an ODK deployment may have. Computer security literature often refers to the “CIA” goals for computer security: confidentiality, integrity, and availability.

1. *Confidentiality.* An adversary should not be able to learn private information about individuals or sets of individuals whose data is collected as part of the ODK system. We can consider varying levels of confidentiality, e.g., it might be OK for some adversaries to learn aggregate information (such as the total number of patients) but not individual information (e.g., the records for a specific person). Confidentiality might also apply to the enumerators (e.g., the healthcare workers)—an enumerator may not want their location or time of data collection disclosed to some adversaries.
2. *Integrity.* An adversary should not be able to cause false information to be collected or stored as part of the ODK application. These adversaries might include enumerators trying to avoid doing their work, beneficiaries lying to enumerators, application developers surreptitiously modifying data after collection, and so on. Guaranteeing that false information is never collected may be difficult or impossible in general. An alternate version of this goal may be: it should be possible for the managers of the deployment or the data analysts to detect and/or mitigate discrepancies due to false data collection.
3. *Availability.* Data, and the ability to collect data, should remain available even if a device is disconnected from the Internet for an extended period of time, or if the device is lost or stolen. Remote access to servers should be robust to denial-of-service attacks.

**Potential adversaries.** We next identify potential adversaries and adversarial goals to an ODK deployment. Potential adversaries include any stakeholders of the deployment itself, as well as external actors. For example:

• *Enumerators* may provide fake data in an attempt to simplify their own jobs, or may violate the confidentiality of data provided by a beneficiary by disclosing private information (e.g., HIV status) to someone not intended to learn the information (e.g., a spouse).
• A given deployment may involve multiple *partners* who are involved with different parts of the deployment and are intended to have access to different forms and/or data. A malicious partner might violate this intention.
• *Governments or other powerful organizations* may target sensitive ODK deployments (e.g., those collecting information about government-related opinions) in order to learn the identities of or information about stakeholders involved in the deployment.
• *Other adversaries* not targeting the deployment specifically may nevertheless cause problems. For example, external hackers may attack the deployment servers and corrupt or steal the data or take the server offline, or thieves may steal mobile devices involved in the deployment for the hardware, resulting in the loss of data.

**Potential threats.** Finally, we consider concrete threats that may result from the above adversaries. For example:

• Unauthorized access to forms or data on the device, or to the remote server, to access, modify, or delete data
• Entering fake data into a form
• Coercing or bribing enumerators or other deployment stakeholders to reveal sensitive information about the deployment or beneficiaries
• Physical theft of a mobile data collection device
• Legal access to data, e.g., through a subpoena
• Inability to use the data collection application
• Fake ODK applications on software marketplaces
• Improper disposal of devices used in data collection
• Other malicious applications installed on devices
• Information leaking to other applications on the device
• Denial-of-service attacks preventing data from being uploaded to the server

We know some of these threats are not solely hypothetical. For example, by default ODK data is stored in plaintext on the SD card; this data can easily be extracted from the device and is world-readable in some versions of Android.

### 4.2 Possible defenses

We now turn to possible defenses. Some of these defenses are already available to ODK deployment architects, some embody standard best practices but have not been incorporated into ODK yet, and others employ either new ideas or ideas from the computer security literature.

**Available defenses and best practices.** Existing security measures supported by ODK or otherwise available on Android include:

• Encryption of saved ODK data
• Encryption of the Android device
• Encrypted connection to the server (i.e., TLS/SSL)
• Access control for server access
• Android apps to lock down phone capabilities
• Checks to prevent or detect fake data entry
• Locking the phone screen
• Keep software up to date

**Additional defences.** We considered a set of possible additional defenses. We report here on those that we later discussed with participants because we thought they had the most potential to be relevant to a variety of deployments. These additional defenses include panic passwords, geographic restrictions, and dongles to replace or augment passwords. Panic passwords are passwords that can unlock a device but simultaneously trigger an alert, erase data, or present synthetic data (Clark and Hengartner, 2008); panic passwords are particularly useful when users might be coerced into unlocking a device against their will. Geographic restrictions refers to the notion of limiting certain functionality, e.g., data collection or access, to specific geographic regions. Using authentication dongles instead of passwords can lead to increased usability, assuming that the user has a dongle in their possession; using dongles in addition to passwords can provide greater security than passwords alone.

## 5 Methodology

In Section 4 we surfaced a spectrum of threats and security considerations potentially applicable to ODK-based deployments. However, as noted earlier, not all threats are equally likely nor have the same impact. Some threats may never manifest because they are either too costly for an adversary or the rewards to the adversaries are too little. Given this nuanced perspective, a key question thus arises: which threats should a deployment seek to mitigate?

This question leads to the second major component of this work: to provide an informed understanding of how ODK users currently perceive the computer security landscape. We designed a two part study—a survey (Section 5.1) followed by in-depth interviews (Section 5.2)—to better understand these issues. We received approval from our institution's human subjects review board to conduct our surveys and interviews. We have also disclosed the results of this work to the core ODK development team, a common practice in security to ensure that developers can react to system vulnerabilities that may be exposed through research.

### 5.1 Survey

We recruited participants through emails to two public mailing lists of ODK developers and community. The survey was open for a period of five weeks. We expect that the survey took around 30 min to complete. We asked participants to provide answers corresponding to a single deployment that they were most comfortable answering questions about, even if they had been involved in multiple projects that used ODK. We did not collect demographic information such as gender, race, or citizenship. We received 56 submissions. All participants reported that they use mobile devices for data collection and use at least one ODK tool.

Survey questions addressed a variety of topics including: type of data; domain of data; size and length of deployment; defenses considered or used; security concerns; incidents of data or device loss, theft, and/or compromise.

### 5.2 Interviews with ODK users

To dive deeper into specific aspects of deployments, we conducted in-depth follow-up interviews with survey participants. 33 of the 56 survey respondents submitted their email address, giving us permission to follow up with them for a phone interview. We contacted all of these people via email to set up interviews. We conducted in-depth interviews with the 10 participants with whom we were able to schedule an interview at a mutually feasible time. Most of the people interviewed were in the “deployment architect” role, but some may take on more than one role. While interviewing beneficiaries, enumerators, or donors would provide additional perspectives on security, we leave this for future work and instead scope this work to those involved with administering the deployment. We chose to focus on deployment architects because they typically have both a broad view of the deployment and direct involvement.

Topics covered included: the participant's role in the deployment, stakeholders or other people involved in the deployment, the purpose and organization of the deployment, what type of data is collected and whether any of it might be considered sensitive, and possible security or privacy concerns or other issues that might have arisen during the deployment. We also elicited from participants their attitudes toward the defenses described in Section 4. These 1-h interviews were conducted via Skype or phone and were audio recorded with participants' consent. Participants included two women and eight men. The deployments spanned several data domains (some more than one): two agricultural, five medical, seven humanitarian, and five in other domains. Nine of these deployments were in developing regions. To protect participant privacy, we did not collect demographic information and we are careful in our presentation of the results to avoid leaking identifying information about participants or their deployments. At a high level, however, participants differed across a number of traits. For example, they included both those operating in rural or urban environments, as academics or humanitarians, working with religious and secular organizations, and embedded in the organization or employed as consultants.

**Interview analysis.** Two researchers were present for almost all interviews and took written notes, which they shared with other group members. Other researchers listened to some of these interviews, took additional notes, and looked at the notes before meeting as a group. As a group, we did an affinity diagramming exercise to come up with a list of themes. The same two researchers independently listened to the recordings and took structured notes based on the themes that were identified in the affinity diagramming exercise. These notes were then checked for agreement before reporting any results. Note that some participants' opinions may have been inconsistent within an interview—these inconsistencies were noted when observed, and interviews were revisited when researchers' notes were inconsistent to determine whether the researchers disagreed or the participant had mixed feelings about a specific topic.

## 6 Survey findings

Before designing our in-depth interviews, we conducted an exploratory survey. Its findings helped us formulate topics and participants for further investigation in interviews.

**Overview.** Most of the 56 respondents collected data in at least one of three data domains: medical (21), humanitarian (22), and agricultural (18). Many categorized their data in multiple ways: 25/56 collect data in more than one domain. A majority (40/56) of respondents reported using paper for data collection in the past. As we discuss in subsequent sections, the switch from paper to digital data collection may influence how people think about data security.

**Sensitive data**. We expected to find a correlation between the domain about which data is collected and whether that data is considered sensitive by the survey respondent. Instead, we find that respondents *across* data domains consider some of the data they collect to be sensitive. Of 55 respondents who answered the question, 36 reported collecting sensitive data, spread across data domains: 16/21 in medical, 18/22 in humanitarian, and 11/18 in agricultural.

**Security risks and incidents.** Our survey asked respondents about whether their deployments had encountered particular security or other incidents, such as lost or stolen data or devices. 17 respondents reported lost (14) and/or stolen (9) devices. Though a lost or stolen device could result in compromised data (since there are ways to access the data once one has access to the physical device, as discussed in Section 4), respondents did not necessarily equate device loss with data compromise: only 7 of the 17 respondents who reported lost/stolen devices also reported that data had been leaked, stolen, or that they didn't know. This mental model may be reasonable—for example, hardware thieves are not necessarily seeking the data on the device—or it may represent a misconception in some cases. One of the goals of our subsequent interviews was thus to learn more about these kinds of incidents, and participants' perceptions of them.

When we asked explicitly about lost or compromised data—which represents direct knowledge of a security incident—9 respondents reported that they had lost data, 2 reported a data leak, and 2 reported stolen data. These results were interesting to us because they suggest that ODK deployments *do* face security threats in practice. We investigate these issues in more depth in our interviews.

We also find that 29/56 respondents report that devices are shared by more than one person in their deployment. Device sharing can pose a risk in some situations because it means that data collected by one individual could be available to other individuals or that the device's behavior (e.g., security settings) might change between users in unsuspecting ways.

## 7 Interview findings

We now turn to our semi-structured interviews. These interviews allowed us to dive more deeply into issues raised in the survey, to provide concrete ICTD-specific perspectives to our threat model, and to gain a more complete understanding of participant attitudes toward security. In Section 7.1 we explore how the priorities of respondents relate to the CIA security goals (confidentiality, integrity, and availability). Then in Section 7.2 we describe how threats related to these concerns can manifest. Next in Section 7.3 we provide examples of actions respondents have taken or considered to mitigate these threats. Finally (Section 7.4), we detail pieces of the broader context influencing these threat models that surfaced during interviews.

### 7.1 Concrete security goals

Our survey results in Section 6 found that many respondents collect sensitive data (including 8/10 interview participants), and our security analysis in Section 4 surfaced interpretations of what “sensitive” might mean. We use our interviews to understand more concretely what participants mean by “sensitive” as well as their broader security goals. We find that data availability and data integrity are prominent goals for ODK deployment architects, whereas confidentiality is not; however, when prompted to threat model, ODK deployment architects do identify reasons for which confidentiality can be important in ICTD contexts.

**Data loss (availability).** For several participants (6/10), a main concern related to the potential derailment of their deployment was data loss. To ODK deployment architects, data loss refers to collected data becoming permanently *unavailable* (and not to the computer security concept of data loss prevention, or the exfiltration of confidential data). P1, when asked about the greatest threat that could derail a deployment, responded “far and away it's data loss. It's just losing the data somehow,” going on to say “I don't worry about somebody getting a copy of the data, I just worry about getting the original data from the remote enumerator into a centralized database.”

In one case the possibility of data loss outweighed the perceived value of security features like encryption, which would support higher *confidentiality* of data:

P2: [Encryption] did cause us some problems and that's why we didn't continue it …. You try to submit some data, some of them get lost along the way somehow.

**Erroneous data (integrity).** Another significant security goal that deployment architects identified was the protection against the entry of falsified data by enumerators. 6/10 participants indicated that they know they have received false data in the past. This goal is a specific example of a data *integrity* goal, against a specific class of adversaries. From the interviews, we learned that enumerators might fabricate data for several reasons, such as avoiding travel to interview locations or shortening interviews by answering “no” to questions that lead to long subsections of a form. P5: “Enumerator fatigue is just as real as [beneficiary] fatigue.”

**Exploited data (confidentiality).** Compared to data loss and erroneous data, participants seemed significantly less concerned about data *confidentiality*. When asked to threat model possible ways in which unauthorized parties might access data and what their goals might be, participants identified a number of *hypothetical* consequences of data breaches:

• Loss of job, e.g., for beneficiaries
• Loss of life or threats of physical harm
• Theft or looting, e.g., knowing a place is vulnerable
• Embarrassment in front of donors

Several of these consequences fit under the broader umbrella of avoiding harm to beneficiaries—a risk that ODK development architects seemed particularly concerned about, when asked to ponder the impact of data disclosures.

Although the impact of a data breach can be greater than the impact of lost or erroneous data, the overall risk of such a breach might be less (since, presently, adversaries do not seem to be intentionally trying to breach the confidentiality of the data collected in these deployments); deployment architects we interviewed seemed to have informally come to this conclusion. Consequently, participants did not consider the potential of data breaches to warrant additional protection beyond their current practices. Moreover, we asked all participants if there was any data that they were interested in but did not collect for computer security reasons; the answer was universally no. Despite the low risk, data breaches are also not hypothetical; P3 reported that a beneficiary lost a job when similar data collected in a previous (non-ODK) deployment was reported to their supervisor.

### 7.2 How threats could manifest

Having now mapped from the abstract security goals in Section 4 to concrete instantiations, we turn to studying concrete threats against ODK deployments.

**Hardware theft (or loss).** Theft of devices was one of the threats that participants thought was the most likely to happen in their deployment. This threat is not hypothetical. Recalling Section 6, 17/56 survey respondents reported lost or stolen devices. In our interviews, three reported having devices stolen, and one participant seemed surprised that no devices had yet been stolen in their deployment.

However, echoing our survey findings, data *confidentiality* was not thought of as the primary concern if a device was stolen or lost. Instead, security of physical devices was approached as a separate issue from data security by most participants. For example, one participant indicated:

P2: We're hoping it's just about the hardware, that's fine. but I don't think it could be an issue about the data inside the tablet. … It's kind of fine, like “take it, reset it, don't look at the data, and enjoy the tablet.”

Despite the known potential for device loss or theft, not all participants considered device loss or theft a serious risk; some thought that the devices were expendable.

**Enumerators as adversaries.** Enumerators are recruited using a variety of strategies, ranging from organization employees to anonymous volunteers with no organizational affiliation. Whereas most enumerators likely have no ill intent, participants identified a number of ways that an enumerator could become an adversary:

• Selling data being or coerced to leak data
• Fabricating data to avoid some part of their job (e.g., travel to a possibly dangerous location, fill out a long boring questionnaire, ask uncomfortable questions)
• Make honest mistakes with data entry
• Accidentally download malware or use excessive amounts of data for non-work activities
• Not caring for the hardware properly/sufficiently

All of the people we talked to have concerns about the veracity of the data being collected by these enumerators, which relates to the data integrity goal. Six were aware of enumerators entering fake data in the past. In at least one case, an enumerator was fired upon clear evidence that he knowingly and intentionally submitted fake data.

Participants noted both technical and non-technical methods that an enumerator could use to compromise the privacy of beneficiaries. There was skepticism that enumerators have the skill to mount even modestly sophisticated technical attacks. However, P3 noted the feasibility of non-technical attacks. The easiest way to gain access to the data might simply be to talk to the enumerator that conducted the interview: “it would be much easier to bribe or go and see an enumerator and offer him a beer.” A key lesson is threats may exist regardless of the technical defenses.

**Weak vs. powerful adversaries.** Most of the attacks we have discussed so far would be carried out by adversaries with limited technical skill and resources. Although these weak adversaries can pose threats, a powerful and motivated adversary can introduce a wider range of threats and potential attacks. For example, if an attacker does not know how to copy data off of a device, the device may be much safer than paper, which can be read by anyone. For a stronger adversary with technical skill, however, digital data could be duplicated without leaving evidence of a data breach.

The most explicit example of a powerful potential adversary came when one respondent indicated that their data, when viewed in aggregate, could reflect a group perceived as dangerous in a negative light, stating:

P2: We record violence that those children might have went through walking on the street. And actually it turned out that the highest perpetrator is, well I cannot mention the name now, but … it's a very dangerous group. We still collect it on the tablet but we don't give out this information to anyone because it will put everyone at risk, whether us or those children, everyone in the program will get in trouble if we give out who is the highest percentage of the perpetrator … We only give it to the UN agencies if they request it, and then we keep it in conversation, we don't actually email even this information, because if it gets intercepted, again, everyone would be in trouble. So it's not stopping us from collecting the data, it's just sharing the data becomes trickier.

In general, even if they had given consideration to more powerful adversaries, they did not seem to have ideas of how those adversaries might compromise their system.

### 7.3 Mitigations

We now explore the steps participants take or might take to mitigate computer security risks.

**Defending against enumerators faking data.** While most enumerators are likely trustworthy, Section 7.2 identified untrustworthy enumerators as a real threat. Using techniques similar to those in (Birnbaum et al., 2013) and (Birnbaum et al., 2012), 6/10 respondents indicated that they include explicit checks into forms or look for data abnormalities to detect possible fabricated data. These checks include: (1) GPS readings to ensure the enumerator was in an appropriate location when completing the form (2/10), (2) timestamps to measure survey completion time (2/10), and (3) requiring enumerators to take photographs of relevant locations (2/10). In some cases enumerators are informed these checks are monitoring their actions, while in other cases they are not.

However, others noted that these checks do not come without additional costs. In some deployments, there might be a desire not to collect GPS coordinates due to sensitivity or technical constraints (e.g., it takes too long to register a GPS signal). A broader lesson, although common in the computer security literature, is that computer security defenses can come with a cost, and that it can be challenging to balance between the benefits of the defense and the additional costs; in this case, for example, collecting GPS coordinates for all deployments could result in violating the privacy of enumerators (since their precise locations would be known) or harming beneficiaries (if the locations of the beneficiaries are sensitive, and the data were ever to be exposed).

**Defending against non-prescribed device use.** The security of devices can be compromised by malware or non-prescribed uses that could impact the deployment, e.g., by transmitting data to an external source or consuming a data quota. Organizations were aware of this threat and took several precautions to limit certain aspects of device use. 4/10 respondents reported that they employed defenses at the level of the mobile device software, such as an app that limits the functionality supported by a device. For example, the deployment devices might only allow the ODK app and a GPS app to be opened without an administrator password.

However, some participants provided reasons why they would not completely lock down devices. One participant made exceptions based on the context of individual enumerators' duties. Those going into a conflict region, for instance, were given devices that were not as locked down, including access to phone and email apps, to keep them useful if they were to be in danger and require a phone:

P3: I couldn't see myself limiting them from the benefits they could get from the tablet in case they were in this kind of [dangerous] situation. Meaning having access to phone, having access to their emails.

The existence of these exceptions speaks to the tradeoff mentioned earlier: incorporating computer security defenses can have negative consequences, and the benefits of security defenses may not always outweigh those consequences.

**Protecting devices.** Since the risk of losing or breaking a device is real, and since the devices are physically under the control of enumerators, some participants employed mechanisms to help enumerators physically protect their devices. A strategy expressed by at least three participants was to confer liability of the tablet (e.g., cost) to the enumerators, believing that this would cause enumerators to taking greater care with the tablets. Some gave the participants protective cases along with devices, used plastic containers to transport devices, or locked devices in cabinets.

Participants also described what they do after a device has gone missing. One survey respondent mentioned tracking down the device based on GPS location. Others used the phone's remote wipe capability, if the device was able to connect to the Internet. The use of remote wipe suggests some concern over protecting the confidentiality of collected data though, as noted in Section 7.1, confidentiality did not emerge as a goal on the forefront of participants' minds. Indeed, many of other precautions employed by participants to protect devices (e.g., making enumerators financially responsible or locking devices in cabinets) speak more to protecting the devices themselves, not protecting their data.

The need to protect devices was not, however, universally recognized, with multiple participants downplaying the importance of protecting devices. One such participant reported zero devices stolen and only a single device damaged. P1 reported never having a device stolen, and reported believing that the “the job itself is more valuable” to enumerators than tablets, going on to say of device theft: “I think that it’s a baseless worry”.

**Backend choices.** Although most of the threats discussed so far pertain to data while on a mobile device, the security of data is a consideration when aggregated as well. No participants indicated that they are concerned about accidental or malicious modification of data once it is stored centrally. Participants expressed varying degrees of understanding about the security guarantees of their data backends. P6 was aware of avenues for data accessed once the data was on a server that could have implications for confidentiality. P3 reported that they would have liked to determine how frequently their partners were viewing the data, as well as to know if the data had been downloaded.

Data is moved to at least one external hosting site in 8/10 deployments. One participant indicated familiarity with the security guarantees of their hosting company:

P2: They say that everything is secure and that their servers … [are] underground [providing] maximum security, no one can infiltrate their data.

Moreover, in this case the participant seemed to delegate security decisions to the hosting company, trusting the hosting company to be secure rather than encrypting their data. Delegation of security responsibilities to other entities may, however, not always be warranted.

**Security through obscurity.** Some participants felt that digital data collection provides some measure of security through obfuscation. To access data on the device requires technical skills and/or knowledge of where and how data is internally represented. An adversary with these skills might be able to duplicate or modify collected data without leaving any trace of their activities. Generally, however, participants were not concerned about this type of attack. For example:

P5: If someone's really interested in the content, it's easier to steal a stack of papers than it is to steal … the technical maze you need to actually make sense of the data. So relative to someone on the ground being able to steal the data content, tablets are much more secure than paper.

This might have been true for the type of adversaries they were most concerned about, but we point out that these technical skills (as evidenced in an earlier section) can be common depending on the context and the adversary.

**Attitudes toward proposed defences.** In Section 4.2 we discussed several possible defensive directions, including defenses already available within ODK, additional best practices, and new directions. Although no one used encryption, the potential value of encryption was widely acknowledged. Participants did identify challenges to using encryption. For example, P2 indicated debugging data loss was more difficult when encrypted: “at what point something went wrong—I was not able to figure that out”.

As a negative result, and contrary to our expectations, no other defense was seen as potentially useful by a majority of participants. Concerns with these defenses were based on (1) (perceived) difficulty of setup, (2) difficulty of use (for enumerators), (3) sufficiency for solving the problem, and (4) monetary cost. As a concrete example, P6 believed that password-based protection would not be effective due to the practices of enumerators. “[Passwords] might not work with people because … I will give it to you because I know you.” Geo-based security would not work for P3 because there is no GPS data available for the sites they are surveying, making GPS impossible to configure. P5 reported that “sometimes the GPS just doesn't work on these devices.”

### 7.4 Broader context

Finally, stepping back, we discuss several factors participants surfaced that influence their knowledge, mental models, and actions surrounding computer security.

**Roles and responsibilities.** An important factor in what technical defenses are considered or implemented is how much responsibility the deployment architect takes for determining the appropriate security mechanisms.

P7: You know the company in their mind, they would keep saying, “Well, after the pilot is done, you're going to bring the server back to our data center, right?” And I kept reminding them that, look, “Are you guys experts in security? What if someone really got interested in the entire data set? What if they hacked in? Are your IT guys savvy enough?” … It was sort of a process, we were trying to encourage them that the cloud is a [more] secure environment for them.

Another participant cited a lack of technical expertise as an explanation for their decisions to make weaker security-related decisions:

P8: And the local server also has several passwords, and the password that I'm using for the local servers is probably not as secure as it should be. I'm not really an IT guy, and as you may know setting up a local server is complex and a bit finicky. It's very easy that it doesn't work, and it's hard in my experience troubleshooting getting it working right, so I've tried to keep things as simple as possible, and it works but it could be more secure.

As we discuss further in Section 8, the dispersal of responsibility for computer security decisions among different stakeholders in a deployment, and among people with different degrees of technical expertise, affects the security-related decisions and tradeoffs that are made.

**Ethics board considerations.** Another group that may (or may not) be responsible for enforcing secure practices is the Institutional Review Board (IRB). Most participants considered ethical issues related to their deployments, and three reported experience with an IRB. These three participants had experiences with IRBs in more than a single country and stated that the IRB process and requirements varied widely between countries:

P1: There are not universal requirements for the IRB … It's kind of wild west stuff actually. There's no universal requirements … They will put requirements that are specific to that piece of research so it depends on very much on the area.

This variation may be desirable: P3 mentioned that an in-country IRB supported work that was relevant and acceptable in the local context but that may have seemed problematic to foreign IRBs. However, weak IRB requirements can also have negative security consequences for deployments. Since the IRB is in a role of requiring compliance with its policies, deployment architects may defer to those requirements. IRBs, however, may lack the technical expertise (or otherwise fail to) require specific security practices:

P1: [Encryption is] something [the IRB] should be requiring and … just lack the technical sophistication to ask for it.

As the technical contractor hired for the deployment, this participant did not consider themselves in a position to impose technical requirements on the study, deferring instead to the IRB's (weaker) requirements.

**Community privacy and security norms.** Although our goal was not to understand the subtleties of the communities in which ODK is deployed, local norms may play an important role in threat modeling. For example, although knowledge of what method of birth control a woman uses may not be particularly sensitive data in some contexts, in places with more conservative values data that reveals a woman is using birth control at all (possibly without her husband's or family's knowledge) could put her at significant risk. Similarly, some types of data may be considered less sensitive in local cultures than in the (different) culture of a deployment architect. For example, one participant was surprised by the amount of information shared by a collaborating organization:

P4: The privacy concerns of the schools from my experience are not particularly strong. For example, something that I would consider to be sensitive is they have … information about either special needs or poor households … When we've requested summary statistics from them, we've often received a lot more details than I would expect them to be comfortable sharing. … In general I think, in the rural areas where we mostly work, a lot of these things are kind of treated as common knowledge. Within the village everybody knows who's poor, everybody knows if you have some sort of special needs, so I don't think it's really on the forefront of their minds.

**Sensitive groups.** Five participants considered data they collected about vulnerable groups—such as children, women, refugees, and victims of violence—especially sensitive. One participant voiced this concern emphatically, suggesting that the vulnerability of the population was a larger consideration than the particular type of information collected:

P5: The most sensitive part about it I think that would jump out to an external eye is just the fact that you're working with a vulnerable population, and that population being children. Kind of full stop there. … Yeah, you're collecting data about their weight and their age and stuff, which ultimately isn't sensitive, but you're collecting data about children.

**Historical context: paper forms.** When the technology used in a deployment changes, mental models surrounding that new technology are influenced by mental models about the old technology. In this case, many (9/10) of the participants had previously employed paper forms for data collection, before switching to ODK for digital data collection. In some cases participants still use paper forms for some parts of their data collection. Though there are many axes along which to compare these two technologies, we focus specifically on security issues here. Participants had mixed views about the security of digital data collection as it compares to the security of paper data collection and gave examples both of how they believe digital may be more secure than paper and of how paper may be more secure than digital.

Several key security *advantages* that participants mentioned of digital over paper are based in the fact that data is uploaded to a server (when a network connection is available). This syncing improves data availability—reducing the amount of data lost when a device is lost—and data integrity—allowing rapid feedback to enumerators to improve the quality of their data. There was also a perception that digital data collection improved the confidentiality of data, because it increases the technical barrier to reading data from a device than from a paper form:

P1: Those surveys that were done on paper were openly readable to anybody who had access to the paper. And their privacy is better served by putting the data into a format that is inaccessible. Our enumerators collect all this data, but as soon as they collect it, it's locked away from them. So they can't share it with each other in any way. And they can't review it. They can't do anything. They can record it, but then all they have is their memory of it.

This perception reflects the previously discussed “security through obscurity” mental model exhibited by several participants—that the barrier of technical knowledge is sufficient to protect the data. In the face of a moderately technical adversary, however, the threat to confidentiality may be greater, since that data can be easily queried (unlike searching through many paper forms). Participants generally did not consider such adversaries, however.

Increased confidentiality can also come from the ability to employ technical means to protect digital data:

P1: The digital database provides a kind of guarantee of confidentiality if only because it's controllable and inaccessible and transportable in a way that is controllable. You can password protect every part of it.

On the other hand, a key security *disadvantage* of digital data collection that participants mentioned was that it affords the collection of more, and more sensitive, information:

P1: The only thing that is specifically unique to doing digital data collection is that you have more identifying information, like you have the GPS coordinates of people's homes and you have the times and dates when they were there. … And you could have photographs and audio recordings and all kinds of things … those things are unique to digital data collection.

Finally, there are differences in security perceptions on the part of the people from whom data is collected. One participant mentioned that beneficiaries are (perhaps incorrectly) more trusting of tablet- than paper-based data collection:

P2: [When] you use the tablets … they feel a bit more safe than when you're using the paper forms. … They … trust you more when you're using those tablets because they also assume that it’s going to be more secure than carrying around a bunch of paper forms … But actually if they know how the tablets also work … technically it's still as insecure as paper methods.

## 8 Discussion

Having presented our threat model, survey results, and interview results, we step back and reflect upon their broader implications. These implications are in addition to more specific lessons and recommendations mentioned previously. In particular, we urge readers implementing similar systems or using ODK to revisit the specific defenses and best practices listed in Section 4.2, and to conduct a threat-modeling exercise specific to their individual use-case and context, as described in Section 4.1.

### 8.1 Broader considerations

**Diversity of stakeholders and views on security.** As in other ICTD contexts, our results surface the importance of considering the full spectrum of stakeholders, who may each have different perspectives on computer security. (Indeed, as we consider in our threat modeling process, some stakeholders may also become adversaries.) We summarize several previous examples to underscore the importance of considering this diversity. For example, ethics boards have a sense of breadth that comes from their exposure to many different projects, but are not necessarily well-versed in specific technical best practices. Meanwhile, the data being collected ultimately comes from beneficiaries, who may have different perceptions about the sensitivity of their own data. External parties do not necessarily understand local context, while locals may not realize how information could be misused outside of that context. These differing perspectives must be carefully considered for each deployment. The diversity of stakeholders and stakeholder views on computer security means that there may not be a “one size fits all” solution for computer security for ICTD systems.

**Challenges with diffused responsibility.** A consequence of the diversity of roles and responsibilities within a deployment, also surfaced in Section 7, is a perceived (or actual) dispersal of responsibility for security. This can lead to an environment where no one feels they are able to intervene with what they consider best practices. This surfaced in multiple ways. For example, one participant was thinking about security but felt unable to act because their development contract did not ask for security defenses. As another example, in their description of how their system uses a cloud hosting company, a participant delegated security responsibilities to the hosting company rather than consider the system holistically. Even if security decisions are consolidated to a designated person within an organization, an additional challenge is that many actors may still have a role in implementing the chosen security defenses, ranging from enumerators to development architects.

**Considerations for threat modeling.** Deployments are more likely to be secure if, before data collection begins, an organization considers how data might be used and who might want it. As part of our interviews, we invited participants to create threat models for their deployments. Some had already begun this process, but only one had done it formally. We believe that this process is valuable, because even if no new or realistic threats are uncovered, it is important to make security choices grounded in a thorough understanding of the tradeoffs rather than in an ad hoc manner.

A common theme among our interviews was the perception that the relative technical sophistication required to access digitally collected data made it more secure. Though many of the realistic threats and adversaries considered by participants may indeed be thwarted by the need for technical expertise, we caution deployment architects to consider more sophisticated adversaries as well.

In particular, technologies, threats, and adversaries may change over time, so threat models must be periodically reevaluated. Collected data may be retained for a long time, and in that time it is possible that new attacks will emerge that make it easier to access that data and/or that the data may be used or combined in unanticipated ways. Even if an attacker's capability does not evolve, the value of a beneficiary's data may change over time (e.g., as a child grows up and enters politics), increasing the willingness of an attacker to put forth technical effort to carry out an attack. Section 7 discussed how a transition from paper to digital data collection has already affected threat models, emphasizing that threat modeling must be an ongoing process.

**ICTD security can leverage traditional security.** In studying computer security for data collection systems in ICTD, we find that, broadly speaking, the challenges to implementing computer security in an ICTD context echo challenges that are well known in non-ICTD contexts. For example, both contexts face tradeoffs when attempting to integrate security with other (usability or functionality) goals, and both contexts can benefit from employing computer security best practices. One difference, however, is that there have been few high profile attacks on ICTD data, and hence ICTD deployments have not felt the same adversarial pressure as other technology domains. We posit that if high profile attacks do emerge, they will transform organizational attitudes toward the likelihood of future external threats.

### 8.2 Recommendations for system designers

Finally, we step back and make recommendations for the designers of systems like ODK.

**Implement defenses to fit current workflows.** Since users of a system must make practical tradeoffs, it is important to design defenses and other security features so that they fit into existing workflows. For example, in Section 7 we found that no participants enable encryption, despite abstractly finding it valuable. Often this decision was made consciously, not accidentally—those who experimented with encryption chose not to use it when they lost data or because they found it made debugging more difficult. In other words, if ODK simply made encryption the default, that would not necessarily increase its use and may harm other deployment goals (e.g., data availability). Instead, features like encryption must be designed in a way that also supports other deployment goals (echoing existing lessons learned in the computer security community, such as that simplicity and usability are crucial to adoption, e.g., (Yee, 2004, Whitten and Tygar, 1999)).

One discussion point is whether it is better to provide no security or some partial level of security. Providing no security could help avoid a situation in which deployment architects incorrectly conclude that using the provided security mechanisms ensure sufficient security under a suitable threat model. Instead, knowing that no security is provided, they could work with security experts to build an appropriately secure system for their deployment. However, one might argue that some security is better than none, especially if deployment architects do not have the resources to work with experts. Without working with such experts, the resulting system would end up with no security. This trade-off is a point that the ODK authors considered when designing ODK (ODK Team, 2015). We do not take a specific stance on this issue, but note that there are valid arguments on both sides.

**Support auditing of device use and data.** Increasing the extent to which systems can be audited would address concerns about data integrity (Sections 7.1, 7.2). Logging mechanisms could detect non-prescribed use (Section 7.3) but allow non-prescribed actions, like phone calls, in emergencies, compared to phone locking applications which cannot make exceptions without a password. Records of when and how often data was viewed, both on the device and in the cloud, can reveal access patterns that might indicate inappropriate curiosity or malicious intent. Similarly, one participant suggested that recording when a screenshot was taken would be useful to indicate that data may have been inappropriately captured for distribution.

**Consider the broader technical ecosystem.** In addition to considering human factors of a threat model (e.g., different perspectives on security), it is important to consider the broader technical ecosystem in which an application may be used. For example, malicious applications may be installed on the same device as a digital data collection application like ODK, suggesting that designers should be cautious about data they write to world-readable locations on the device. Additionally, system designer may rely on external components for certain functionality: for example, QR codes—as used by one participant—may be read by an external application; indeed, ODK developers recommend using a third-party application to scan QR codes. System designers must include these external components as part of their threat models. For example, some QR code applications may transmit QR codes or GPS coordinates to their backend systems, which may violate the data flow and data privacy expectations of an ODK deployment architect. Consequently, it may be preferable to implement certain functionality directly into a system rather than relying on (possibly untrusted) external components.

### 8.3 Limitations and future work

The main limitations of this work come from potential participant selection bias. Our selection of deployment architects was limited to those who responded to a survey sent to an ODK mailing list. Subscribers to this mailing list may not be representative of all deployment architects using ODK, and the members of this list who chose to respond to the survey may differ from other members of that mailing list. For example, people monitoring the mailing list may have greater than average technical interest, might have fewer technical skills and subscribe to seek help, or might be more or less likely to belong to certain types of organizations. Deployment architects who elected to respond to a survey about security may have more sensitive data or may have given more thought to security before taking the survey. We do not know how (or if) this recruitment strategy affected our results. Furthermore, interview participants not only responded to the survey but also consented to follow-up conversations. Again, this could have resulted in a group of participants who were more (or less) concerned about the security and sensitivity of their data.

As discussed in Section 5.2, we chose to focus on “deployment architects” rather than other stakeholders involved in deployments. This decision was made because these individuals are the most intimately familiar with technology and have the most direct input into how technology is used. However, security is an organizational concern. Expanding the pool of respondents to include those in different roles, from different organizations, or that might be more or less security conscious could be a productive avenue for future work. Interviewing the beneficiaries that provide personal data, the enumerators that collect data in the field, or the policy makers that influence technological decisions could yield additional insights that were not brought to light by focusing on deployment architects.

Finally, our work did not uncover any active attacks or data breaches. However, that does not mean that such events have not occurred. It is possible that such things have happened or are currently ongoing but that respondents are unaware of them. If such an attack comes to light it will undoubtedly impact threat models and affect how deployment architects approach security.

## 9 Conclusion

Digital data collection is an important activity for many organizations in the developing world. We focused on ODK as a widespread digital data collection platform and conduct a computer security threat modeling exercise to evaluate attacks that could target ODK deployments. We conducted a survey and interviews with organizations using ODK to understand what threat models are considered in the field. Leveraging our threat model, survey, and interview results, we explore the challenges of computer security in digital data collection in an ICTD context and make recommendations to organizations seeking to keep their data secure.

## Footnotes

2 https://securitycards.cs.washington.edu/.
